# Circulating microRNA-155-3p levels predicts response to first line immunotherapy in patients with metastatic renal cell carcinoma

**DOI:** 10.1038/s41598-024-59337-4

**Published:** 2024-04-13

**Authors:** Maryam Soleimani, Marisa Thi, Sajjad Janfaza, Gizem Ozcan, Sylwia Mazurek, Guliz Ozgun, Corinne Maurice-Dror, Bernhard Eigl, Kim Chi, Christian Kollmannsberger, Lucia Nappi

**Affiliations:** 1 Division of Medical Oncology, British Columbia Cancer-Vancouver Cancer Centre, 600 West 10th Avenue, Vancouver, BC V5Z 4E6 Canada; 2 Auckland Cancer Society Research Centre, Faculty of Medical and Health Sciences, University of Auckland, Auckland, New Zealand; 3 Vancouver Prostate Centre, Department of Urologic Sciences, University of British Columbia, Vancouver, BC Canada; 4 Department of Cancer Immunology, Poznan University of Medical Sciences, Poznan, Poland

**Keywords:** Tumour biomarkers, Predictive markers, Renal cell carcinoma

## Abstract

Predictive biomarkers of response to immune checkpoint-based therapies (ICI) remain a critically unmet need in the management of advanced renal cell carcinoma (RCC). The complex interplay of the tumour microenvironment (TME) and the circulating immune response has proven to be challenging to decipher. MicroRNAs have gained increasing attention for their role in post-transcriptional gene expression regulation, particularly because they can have immunomodulatory properties. We evaluated the presence of immune-specific extracellular vesicle (EV) microRNAs in the plasma of patients with metastatic RCC (mRCC) prior to initiation of ICI. We found significantly lower levels of microRNA155-3p (miR155) in responders to ICI, when compared to non-responders. This microRNA has unique immunomodulatory properties, thus providing potential biological rationale for our findings. Our results support further work in exploring microRNAs as potential biomarkers of response to immunotherapy.

## Introduction

Renal cell carcinoma is the most common malignancy affecting the kidneys and is frequently diagnosed in advanced stages. Historically, prognosis has been poor for mRCC, ranging from 8–43 months depending on International mRCC Database Consortium (IMDC) risk stratification^1^. Recent advancements in systemic therapy have led to significant improvements in survival, with multiple first line treatment options now available, as outlined below.

Renal cell carcinoma is a metabolically and immunologically active malignancy, and current first-line systemic therapy options for mRCC include the combination of dual immune checkpoint inhibitors (ipilimumab plus nivolumab), or combination of an immune checkpoint inhibitor plus an oral vascular endothelial growth factor receptor tyrosine kinase inhibitor (VEGFR-TKI; pembrolizumab plus axitinib, nivolumab plus cabozantinib, pembrolizumab plus lenvatinib)^2–5^. While these therapies have been demonstrated to be highly active and result in overall survival improvement, many patients do not derive durable benefit, and are at risk of significant treatment toxicity. Therefore, an important unmet need in mRCC is the development of biomarkers to predict response (and/or resistance) to ICI. Unlike in other tumour sites such as non-small cell lung cancer (NSCLC) and melanoma, tissue programmed death-ligand 1 (PD-L1) score does not appear to be a reliable predictive biomarker of response^6^. In recent years, the utilization of liquid biopsy in the development of highly sensitive and specific oncologic biomarkers has emerged^7–11^.

Extracellular membrane vesicles (EVs) are produced by the exocytosis of intraluminal vesicles into the extracellular space. Circulating EVs interact with various targets, modulating immune function, enhancing tumour invasion, and altering the TME^12–14^. Tumour-derived EVs carry a variety of components, including DNA, microRNAs (miRNAs), and proteins. MicroRNAs are small non-coding RNAs that regulate gene expression^15^. Extracellular vesicle-derived miRNAs can be effectively internalized and functional in recipient cells, confirming that these contents do indeed impact the cells they interact with^16^. More recently, miRNAs have been identified that have unique immunomodulatory properties, but the role of these miRNAs as predictive biomarkers of response to ICI is not well-characterized^17–20^.

In this study, we first undertook a literature review to evaluate miRNAs which have been implicated in mRCC and may have immunomodulatory properties. We then extracted and quantified the levels of these miRNAs from the plasma of patients with mRCC, and from healthy participants using standardized protocols. We hypothesized that the levels of EV miRNAs would vary between responders versus non-responders to ICI and could therefore be proposed as a biomarker to predict response.

## Methods

### Patient selection

All methodology was carried out in accordance with our institutional guidelines. After obtaining University of British Columbia BC Cancer institutional research ethics board approval (REB # H20-00076), we reviewed our provincial genitourinary biobank for patients with histopathologically confirmed RCC who had stage IV disease and had received first line ICI between March 2017 and January 2020 (n = 40). As part of this biorepository for research purposes, we obtain informed consent from participants, after which they provide 30–60 millilitres (mL) of whole blood in Streck tubes prior to initiation of systemic therapy. Patients were excluded if they had an active second malignancy, or if they had withdrawn consent from participation. Clinical data were obtained from the patient record. Consent was also obtained from 30 heathy participants (with no history of malignancy or autoimmune condition) for collection of 30 mL of whole blood to serve as control samples.

### Extraction of miRNAs and evaluation of pathways

Using the miRBase database, miRNAs implicated in RCC and associated with the antitumor immune response were selected (See Supplementary Table S1)^21^.

To ensure that microRNA levels in samples were not impacted by hemolysis, we analyzed hemoglobin concentration by spectrophotometry. Absorbance values of hemoglobin were measured at 406 nm, 414 nm, 431 nm, 541 nm and 576 nm with a baseline correction at 750 nm using a Nanodrop OneC. 2 μL of each sample was measured in triplicates, and ddH2O was used as blanking solution. The concentration of hemoglobin was calculated based on manufacturer’s custom method formula. A cut-off of 5 g/L was used to detect hemolysis. One sample was excluded from analysis due to hemolysis.

MicroRNAs were extracted from 1 mL of plasma of patients with mRCC and healthy volunteers using the exoRNeasy serum/plasma midi kit (Qiagen, Hilden, Germany, ID 217184) and the following Applied Biosystems TaqMan^™^ assays (hsa-miR-1233-3p [assay ID: 002768], hsa-miR-221-5p [assay 002096], hsa-miR-200a [assay ID 001011], hsa-miR-200b [assay ID 002251], hsa-miR-155-3p [assay ID 002287], hsa-miR-424-5p [assay ID 000604], hsa-miR-138-5p [assay ID 002284], hsa-miR-497-5p [assay ID 001043], hsa-miR-520c-3p [assay ID 002400], and hsa-miR-3065-5p [assay ID 242265_mat]).

Reverse transcription (RT) was then performed using a TaqMan microRNA reverse transcription kit (applied biosystems, Darmstadt, Germany, C# 4366596). The RT product was pre-amplified to increase sensitivity (TaqMan^™^ PreAmp master mix kit C# 4384267). The level of each miRNA was measured by real time polymerase chain reaction (PCR) using TaqMan^™^ miRNA assay (applied biosystems). Cel-miR-39-3p (assay ID 78293_mir) was used as spike in control for miRNAs extraction efficiency evaluation. Cycle threshold (Ct) values were normalized to hsa-miR-30-5p (assay ID 000602). The relative quantity (RQ) of the level in the patient samples compared to the healthy volunteer samples was calculated using the 2^ΔΔCt^ method.

The EVs were extracted from 4 ml (mL) of plasma for EVs characterization using a modified version of the exoRNeasy serum/plasma maxi kit (Qiagen, Hilden, Germany, ID 77,164). Before binding of vesicles to the membrane affinity column with binding buffer (XBP), the column was washed with 3.5 mL wash buffer (XWP) and centrifuged at 500 × *g* at room temperature for 5 min. After binding, this process was repeated. Subsequently, 300 μL of elution buffer (400 mM NaCl [Sigma–Aldrich, S3014] + 50 mM Tris [ThermoFisher Scientific, 17926] + 1 mM DTT [ThermoFisher Scientific, R0861], pH 8.0) was applied to the spin column membrane, incubated for 5 min, and centrifuged at 500 × *g* for 5 min at room temperature, to collect the eluted EVs. The size, density, and purity of EV fractions was assessed through nano-tracking analysis (NTA) conducted on a NanoSight LM10 (Malvern Panalytical) using NTA 3.1 software. Before the read, all samples were diluted 1:1000 with phosphate buffered saline (PBS) filtered through a 0.02 µm (µM) membrane to remove nanoparticle background. The NTA assessments were run with a flow rate of 50 μL/min, at 25 °C with a 488 nm (nm) laser/filter. Videos of Brownian motion of nanoparticles were recorded and analyzed.

### Statistics

Statistical analysis was done using SPSS (version 29.0.0.0; SPSS Inc, Chicago, IL, USA) and figures were created using GraphPad Prism version 8.3.1 (Graphpad Software LLC). For statistical analysis, patients were categorized as either responders (R; defined as best radiographic response of either complete response [CR], partial response [PR], or stable disease [SD]; n = 27) or non-responders (NR; defined as radiographically progressive disease; n = 13). Mann–Whitney U test was used to compare the levels of miRNAs, first comparing patients with mRCC versus healthy controls, then between patients with mRCC according to best response to therapy (R vs. NR). A *p*-value ≤ 0.05 was used to define statistical significance.

## Results

### Baseline characteristics

Clinical characteristics are displayed in Table 1. Median age at diagnosis was 59.3 years. Distribution across IMDC subgroups showed that over 80% of cases were categorized as intermediate or poor risk. The most common pathologic subtype was clear cell RCC (n = 30), with five cases demonstrating evidence of sarcomatoid features on pathology. Sites of metastases were most commonly pulmonary (77.5%), nodal (70.0%) and bone (37.5%). The most common first line treatment regimen was ipilimumab plus nivolumab (n = 32), followed by pembrolizumab plus axitinib (n = 5), and avelumab plus axitinib (n = 5). Three patients experienced CR (7.5%), 17 PR (42.5%), and 7 had SD (7.5%). Finally, 13 had evidence of PD (32.5%).

**Table 1:** Demographics.

Variable (n = 40)	Number of patients (%)
Sex	
Male	29 (72.5)
Female	11 (27.5)
Median age at diagnosis (years) (range)	59.3 (26.1–76.0)
Median age at start of systemic therapy (years) (range)	63.5 (26.6–76.2)
Median time from diagnosis of advanced disease to start of systemic therapy (months) (range)	3.0 (0.0–182.0)
ECOG performance status	
0	10 (25.0)
1	20 (50.0)
2	7 (17.5)
3	3 (7.50)
IMDC at start of systemic treatment	
Favourable risk	7 (17.5)
Intermediate risk	19 (47.5)
Poor risk	14 (35.0)
Underwent nephrectomy	
Yes	27 (67.5)
No	13 (32.5)
Subtype	
Clear cell	30 (75.0)
Papillary	2 (5.0)
Chromophobe	2 (5.0)
Sarcomatoid component on pathology	5 (12.5)
Sites of metastases	
Pulmonary	31 (77.5)
Lymph nodes	28 (70.0)
Bone	15 (37.5)
Liver	5 (12.5)
Type of first line systemic therapy	
Ipilimumab plus nivolumab	32 (80.0)
Pembrolizumab plus axitinib	5 (12.5)
Avelumab plus axitinib	3 (7.5)
Best response to therapy	
Complete response	3 (7.5)
Partial response	17 (42.5)
Stable disease	7 (17.5)
Progressive disease	13 (32.5)

### Differential levels of microRNAs are found in patients with metastatic RCC

After successful extraction and confirmation of EVs by size, density and purity characterization, miRNA were extracted (See Supplementary Fig. S1 and Supplementary Videos 1A–H). We compared the relative levels of each miRNA between patients and healthy control samples (Table 2).

**Table 2:** Differential levels of microRNAs between patients and controls.

miRNA	Patients	Healthy controls	*p-*value
**miR-1233-3p**	**1.8535**	**0.8057**	**0.004**
miR-221-5p	0.0395	9.2038	0.114
miR-200			
200a	0.9735	0.8233	0.740
200b	1.0312	1.0339	0.462
200c	5.2128	2.2349	0.235
**miR-155-3p**	**3.6908**	**0.2103**	**0.012**
**miR-424**	**0.5428**	**1.0367**	**0.011**
**miR-138-5p**	**0.2463**	**1.3734**	**0.031**
miR-497-5p	0.1339	5.6184	0.138
miR-520c-3p	1.6117	0.9023	0.1960
**miR-3065-5p**	**0.4122**	**1.3169**	**0.009**

Significantly higher relative levels of microRNAs 1233-3p, 155-3p, and significantly lower relative levels of microRNAs 424, 138-5p and 3065-5p were noted in patients as compared to healthy control.

Significant values are in bold.

**Figure 1:**
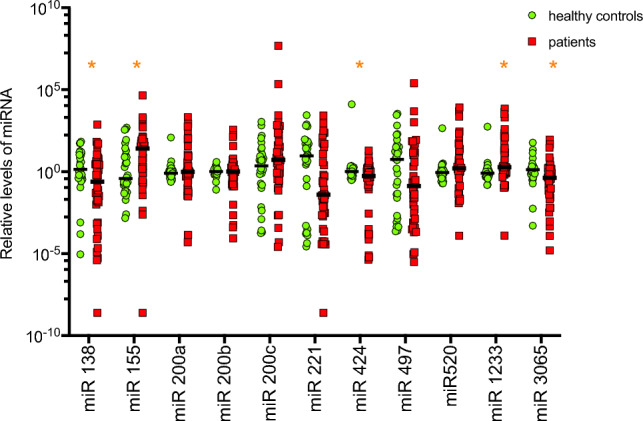
Comparison of the relative levels of microRNAs between healthy controls and patients. Graphic representation of the relative levels of microRNAs in patients with metastatic renal cell carcinoma, as compared to healthy control participants. Asterisks (*) indicates statistically significant differential level. Median levels are indicated by solid black line (–).

**Figure 2:**
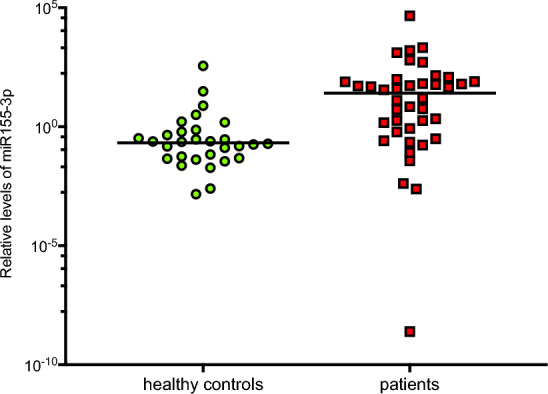
Comparison of the relative levels of microRNA-155-3p in healthy controls versus patients. Median level of microRNA-155-3p was 0.21 in healthy controls, versus 3.69 in patients (*p* = 0.006).

**Figure 3:**
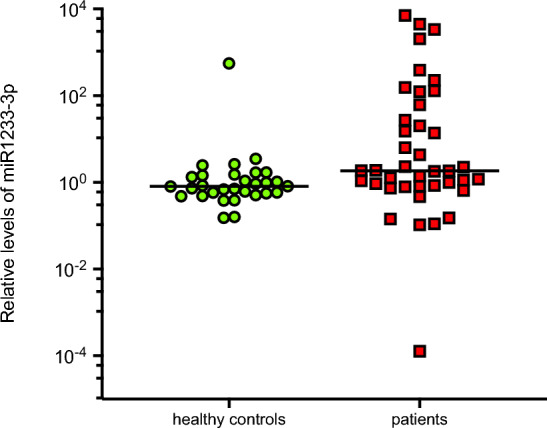
Comparison of the relative levels of microRNA-1233-3p in healthy controls versus patients. Median level of microRNA-1233-3p was 0.806 in healthy controls versus 1.85 in patients (*p* = 0.004).

Patient with mRCC had significantly lower levels of miR-138-5p (0.246 vs. 1.373; *p* = 0.031), miR-424 (0.543 vs. 1.037; *p* = 0.012), and miR-3065-5p (0.412 vs. 1.317; *p* = 0.009), while their levels of miR-155-3p ([miR155] 3.691 vs. 0.210; *p* = 0.012), and miR-1233-3p (1.854 vs. 0.806 *p* = 0.004) were significantly higher (Figs. 1, 2, 3).

### Differential levels of microRNAs correlate with response to ICI

**Figure 4:**
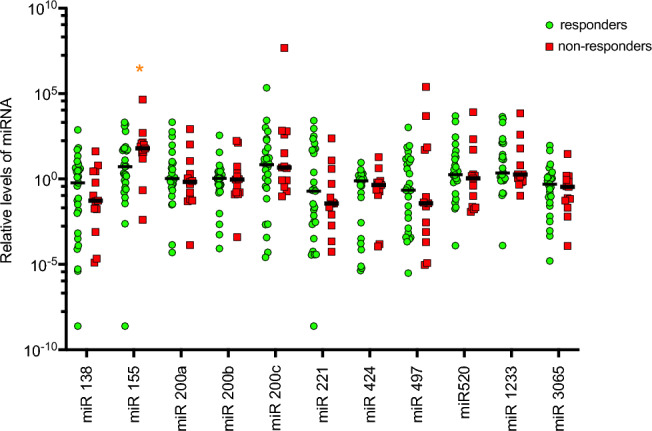
Comparison of the relative levels of microRNAs based on response to immune checkpoint-based therapy. Amongst all microRNAs evaluated, only levels of microRNA-155-3p were significantly different between responders to immune-checkpoint inhibition, versus non-responders (indicated by asterisks *). Responders were defined as those patients who achieved radiographic complete response, partial response, or stable disease, whilst non-responders had radiographically progressive disease. Median levels are indicated by the solid black line.

**Figure 5:**
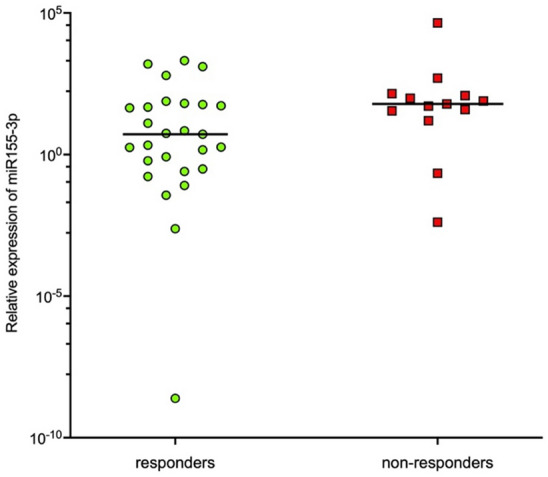
Comparison of the relative levels of miR-155-3p in responders v non-responders. Median level of microRNA-155-3p was significantly lower in responders to immune checkpoint inhibition at 0.62, compared to 35.29 in non-responders (*p* = 0.042).

We then evaluated the same panel of miRNAs within the patient cohort, comparing responders (R) and non-responders (NR). Responders were defined as those patients who achieved CR, PR or SD as best radiographic response to therapy. Amongst those five miRNAs, only miR155 levels were significantly different between R and NR (Fig. 4). The median level of miR155 was 57.9-fold higher amongst NR (35.29 vs 0.61 respectively; *p* = 0.043; Fig. 5).

## Discussion

In this study, we evaluated the presence of immune-specific EV miRNAs in the plasma of patients with mRCC prior to initiation of ICI. We found significantly lower relative levels of miR155 in responders to ICI, when compared to non-responders. Our findings are in keeping with that reported in plasma of patients with melanoma, wherein higher levels of miR155 were seen in patients, as compared to healthy controls, and lower levels of miR155 in patients was associated with improved clinical outcomes^22,23^.

While our results are hypothesis-generating in nature, there is biological rationale for how miR155 could serve as a predictive biomarker of response to ICI. MiR155 is the product of the *MIRHG155* gene, also referred to as the *B-cell Integration Cluster (BIC)* gene, and has often been referred to as the “master regulator of inflammation” owing to its role in modulating the inflammatory response in autoimmune conditions, and oncogenesis^24^. Its most well-characterized role is in negative regulation of activation induced cytidine deaminase (AID), an enzyme that is critical for diversification of the antibody repertoire^25^.

While most of our understanding of the anti-tumour immune response has focused on T-cells, emerging evidence supports an important role for B-cells in this complex process^26^. B-cells are those lymphocytes that express surface immunoglobulins and ultimately lead to antibody production. During B-cell differentiation, several processes take place to allow for diversification of the antibody repertoire; these include AID controlled class switch recombination (CSR) and somatic hypermutation (SHM).

The critical interaction of B and T-cells is also important for achieving tumour control^27^. An important role of B cells is the maintenance of tertiary lymphoid structures (TLS) in the TME. These are collections of B cells, T cells, and dendritic cells, which develop due to chronic inflammation. They function similarly to other lymphoid structures in that they allow for maturation and differentiation of B and T cells in areas adjacent to tumour beds, while also facilitating tumour antigen presentation, and infiltration of lymphocytes within tumours^28^. These TLS have been demonstrated to have prognostic implications in NSCLC, breast, and ovarian cancers^29–31^. Importantly, in RCC, TLS have been demonstrated to produce high levels of IgG, which become bound to tumour cells; the presence of these IgG-bound tumour cells in the TME has been associated with a higher response rate to ICI^32^. Saul and colleagues have demonstrated that in melanoma tissue, not only are unique IgG repertoire subclasses present, but also higher AID mRNA expression is seen within in that tissue suggesting an important role for humoral immune response in TLS^33^. Bod and colleagues recently reported that targeting the B cell surface marker, T cell immunoglobulin and mucin domain 1 (TIM-1) can stimulate antigen presentation and ultimately increase effector T cell activity and an enhanced anti-tumour immune response^34^. This shifting focus to consider B cells as important players in the anti-tumour immune response has led to new advancements to evaluate the spatial arrangement of B and T cells within tissue, with important implication for immune active malignancies^35^.

MicroRNA-155 has multiple regulatory functions and its exact role in RCC is not clear. Lower tissue expression of miR155 has been reported as a poor prognostic biomarker in RCC^36^. On the other hand, miR155 levels have been shown to inversely correlate with VHL expression, such that higher levels of miR155 lead to lower VHL expression and thus promotion of angiogenesis and tumorigenesis^37^. Meng and colleagues reported that higher levels of miR155, both in plasma EVs from patients and in 786–0 and Caki-1 RCC cell lines, resulted in suppressed *forkhead box O3 (FOXO3)* expression and promotion of RCC cell growth^38^. Taken together, these findings emphasize that miR155 is highly relevant in RCC, but further work is needed to clarify its exact role in this malignancy.

Renal cell carcinoma has been well-characterized as a metabolically dysregulated malignancy characterized by increased aerobic glycolysis^39,40^. Much of this is related to upregulation of the hypoxia inducible factor (HIF) pathway due to inactivation of the *von Hippel Lindau* (*VHL*) tumour suppressor gene. Increased HIF activity leads to activation of several metabolic pathways, including those relevant to glycolysis^41^. In breast cancer cell lines, miR155 has been demonstrated to promote tumour cell growth via altered glucose metabolism, by increasing activity of the phosphoinositide 3-kinases (PI3K)-FOXO3a/c-MYC axis, and reducing glucose transport 4 (GLUT4) expression^42^. The latter finding has also been reported in ccRCC, though the link with miR155 has not yet been established^43^. Additionally, the metabolic and immunogenic profiles of RCC are intimately linked within the TME^44^. In fact, as RCC stage advances, changes are noted in tumour infiltrating lymphocytes (TILs), such that they not only display T-cell exhaustion and decreased cytokines production, but their metabolic profile is also altered with decreased expression of metabolically relevant genes, changes in glucose uptake and mitochondrial activity^45^. It has been suggested that metabolic gene expression changes in RCC cells may contribute to ICI resistance^46^.

Our study has several limitations, and the results will need to be validated. First, the small sample size is a limiting factor in being able to make a conclusion that miR155 is in fact a predictive biomarker of response to ICI in mRCC. Validation studies in larger cohorts would be valuable to confirm our finding. Furthermore, miR155 is not specific to RCC and is expressed in other malignancies, inflammatory conditions, and normal tissue as well. We overcame this limitation by selecting patients and healthy control participants who did not have a history of other malignancy or autoimmune conditions. The retrospective nature of our study is a further limitation and as previously mentioned, prospective evaluation in a larger cohort of patients is needed. This is particularly important as treatment may alter the metabolic profile of the tumour, exhaust T cells, and modulate the presence of tumour neoantigens thus impacting the immune response. Finally, while the use of hsa-miR-30-5p for normalization may be considered a limitation given reported expression of this miRNA in RCC, we overcame this by evaluating the levels of this miRNA in the populations of interest of this study. When evaluating both healthy controls compared to mRCC patients, and then amongst patients comparing R versus NR to ICI, no statistically significant difference in hsa-miR-30b-5p levels was observed amongst any groups (*p* = 0.641 and *p* = 0.608, respectively).

Our study adds to a growing body of evidence that is exploring the humoral immune system in the anti-tumour immune response. As questions arise regarding resistance to ICI and there are expanding indications for these drugs throughout stages of disease, EV miRNAs hold promise as potential biomarkers of response to immunotherapy in RCC and should be explored further.

## Supplementary Information

Supplementary Information.

Supplementary Video 1.

Supplementary Video 2.

Supplementary Video 3.

Supplementary Video 4.

Supplementary Video 5.

Supplementary Video 6.

Supplementary Video 7.

Supplementary Video 8.

## Data Availability

The datasets used and/or analysed during the current study are available from the corresponding author on reasonable request.
