# Author Correction: Circulating microRNA-155-3p levels predicts response to first line immunotherapy in patients with metastatic renal cell carcinoma

**DOI:** 10.1038/s41598-025-99376-z

**Published:** 2025-04-28

**Authors:** Maryam Soleimani, Marisa Thi, Sajjad Janfaza, Gizem Ozcan, Sylwia Mazurek, Guliz Ozgun, Corinne Maurice-Dror, Bernhard Eigl, Kim Chi, Christian Kollmannsberger, Lucia Nappi

**Affiliations:** 1 Division of Medical Oncology, British Columbia Cancer-Vancouver Cancer Centre, 600 West 10th Avenue, Vancouver, BC V5Z 4E6 Canada; 2 Auckland Cancer Society Research Centre, Faculty of Medical and Health Sciences, University of Auckland, Auckland, New Zealand; 3 Vancouver Prostate Centre, Department of Urologic Sciences, University of British Columbia, Vancouver, BC Canada; 4 Department of Cancer Immunology, Poznan University of Medical Sciences, Poznan, Poland

Correction to: *Scientific Reports* 10.1038/s41598-024-59337-4, published online 13 April 2024

The Supplementary Information published with this Article contained an error. In Supplementary Figure S1, panel f was a duplication of panel e. The original Supplementary Information file is provided below.

This error has now been corrected in the Supplementary Information file that accompanies the original Article.

## Electronic supplementary material

Below is the link to the electronic supplementary material.

Supplementary Information.

