# Supplementary material for: Circulating microRNA-155-3p levels predicts response to first line immunotherapy in patients with metastatic renal cell carcinoma
Source: Sci Rep. 2024 Apr 13;14:8603. doi: 10.1038/s41598-024-59337-4 (PMC11016103; doi:10.1038/s41598-024-59337-4)
Supplement: Supplementary file 1 — Supplementary Information. [file 41598_2024_59337_MOESM1_ESM.docx]

**SUPPLEMENTARY FIGURES**

Supplementary Table S1: specific miRNA which were implicated in renal cell carcinoma.

| **Specific miRNA** | **Activity** | **Reference** |
| --- | --- | --- |
| miR-1233-3p | Regulation of p53 gene expression | 1 |
| miR-221-5p | Regulation of the EGFR pathway, modulation of dendritic cell maturation | 2-4 |
| miR-200 | PD-L1 expression regulation, development of RCC metastases | 5-7 |
| miR-155-3p | Regulation of T-regulatory cell activity, regulation of AID, regulation of PD-L1 expression, regulation of VHL expression, potential targeting of FOXO3 | 8-12 |
| miR-424 | Anti-tumour immune response | 13 |
| miR-138-5p | Immune checkpoint regulation of CTLA-4 and PD-1 | 14-15 |
| miR-497-5p | Associated with upregulation of PD-L1 in mRCC | 16 |
| miR-520c-3p | NK cell activity, IL8 regulation | 17-18 |
| miR-3065-5p | Angiogenic modulation in RCC | 19 |

Supplementary Table S1 References:

1. Wulfken, L. M. *et al.* MicroRNAs in Renal Cell Carcinoma: Diagnostic Implications of Serum miR-1233 Levels. *PLoS ONE* 6, e25787 (2011).
2. Fu, Y. *et al.* Myrothecine A modulates the proliferation of HCC cells and the maturation of dendritic cells through downregulating miR-221. *Int. Immunopharmacol.* 75, 105783 (2019).
3. Teixeira, A. L. *et al.* Higher circulating expression levels of miR-221 associated with poor overall survival in renal cell carcinoma patients. *Tumor Biol.* 35, 4057–4066 (2014).
4. Dias, F. *et al.* Plasmatic miR-210, miR-221 and miR-1233 profile: potential liquid biopsies candidates for renal cell carcinoma. *Oncotarget* 8, 103315–103326 (2017).
5. Saleeb, R. *et al.* The miR-200 family as prognostic markers in clear cell renal cell carcinoma. *Urol. Oncol. Semin. Orig. Investig.* 37, 955–963 (2019).
6. Chen, L. *et al.* Metastasis is regulated via microRNA-200/ZEB1 axis control of tumour cell PD-L1 expression and intratumoral immunosuppression. *Nat. Commun.* 5, 5241 (2014).
7. Li, Y. *et al.* MicroRNA-200b is downregulated and suppresses metastasis by targeting LAMA4 in renal cell carcinoma. *EBioMedicine* 44, 439–451 (2019).
8. Kong, W. *et al.* Upregulation of miRNA-155 promotes tumour angiogenesis by targeting VHL and is associated with poor prognosis and triple-negative breast cancer. *Oncogene* 33, 679–689 (2014).
9. Yee, D., Shah, K. M., Coles, M. C., Sharp, T. V. & Lagos, D. MicroRNA-155 induction via TNF-α and IFN-γ suppresses expression of programmed death ligand-1 (PD-L1) in human primary cells. *J. Biol. Chem.* 292, 20683–20693 (2017).
10. Dorsett, Y. *et al.* MicroRNA-155 Suppresses Activation-Induced Cytidine Deaminase-Mediated Myc-Igh Translocation. *Immunity* 28, 630–638 (2008).
11. Dudda, J. C. *et al.* MicroRNA-155 Is Required for Effector CD8+ T Cell Responses to Virus Infection and Cancer. *Immunity* 38, 742–753 (2013).
12. Ji, Y. *et al.* miR-155 augments CD8 ^+^ T-cell antitumor activity in lymphoreplete hosts by enhancing responsiveness to homeostatic γ _c_ cytokines. *Proc. Natl. Acad. Sci.* 112, 476–481 (2015).
13. Xu, S. *et al.* miR-424(322) reverses chemoresistance via T-cell immune response activation by blocking the PD-L1 immune checkpoint. *Nat. Commun.* 7, 11406 (2016).
14. Wei, J. *et al.* MiR-138 exerts anti-glioma efficacy by targeting immune checkpoints. *Neuro-Oncol.* 18, 639–648 (2016).
15. Liu, Y. & Qu, H. miR‐138‐5p inhibits proliferation and invasion in kidney renal clear cell carcinoma by targeting SINA3 and regulation of the Notch signaling pathway. *J. Clin. Lab. Anal.* 35, e23766 (2021).
16. Qu, F. *et al.* MicroRNA-497-5p down-regulation increases PD-L1 expression in clear cell renal cell carcinoma. *J. Drug Target.* 27, 67–74 (2019).
17. Min, D. *et al.* Downregulation of miR-302c and miR-520c by 1,25(OH)2D3 treatment enhances the susceptibility of tumour cells to natural killer cell-mediated cytotoxicity. *Br. J. Cancer* 109, 723–730 (2013).
18. Tang, C.-P., Zhou, H.-J., Qin, J., Luo, Y. & Zhang, T. MicroRNA-520c-3p negatively regulates EMT by targeting IL-8 to suppress the invasion and migration of breast cancer. *Oncol. Rep.* 38, 3144–3152 (2017).
19. Müller, S. & Nowak, K. Exploring the miRNA-mRNA Regulatory Network in Clear Cell Renal Cell Carcinomas by Next-Generation Sequencing Expression Profiles. *BioMed Res. Int.* 2014, 1–11 (2014).

Supplementary Figure S1:

A B


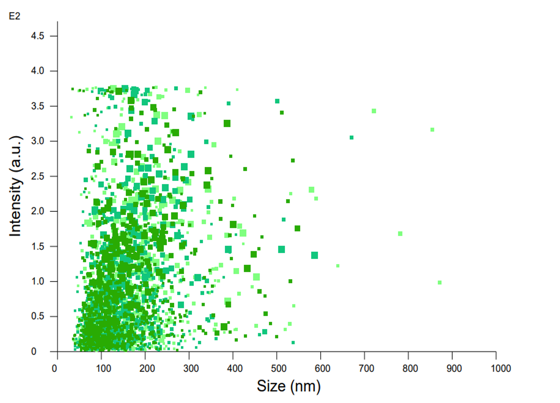

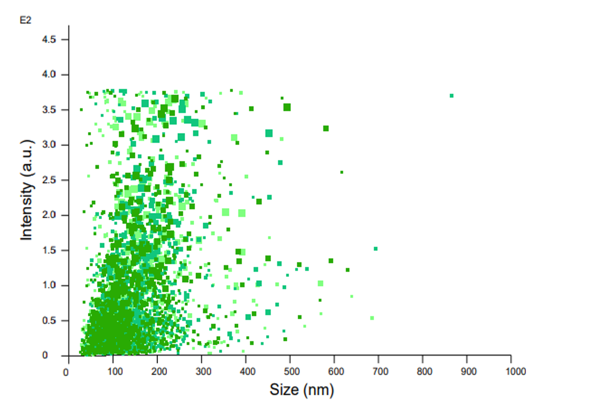


C D


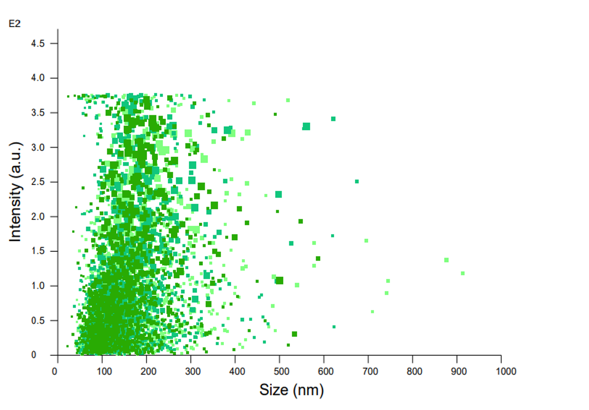

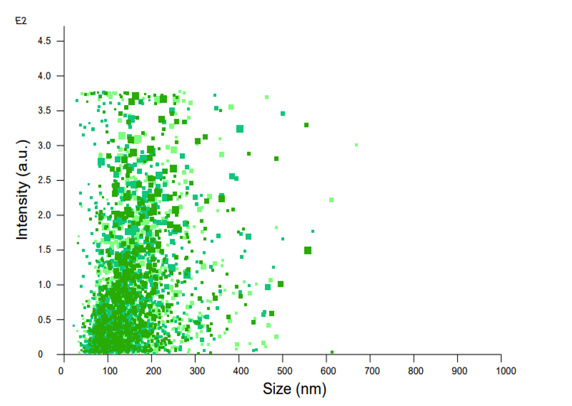


E F


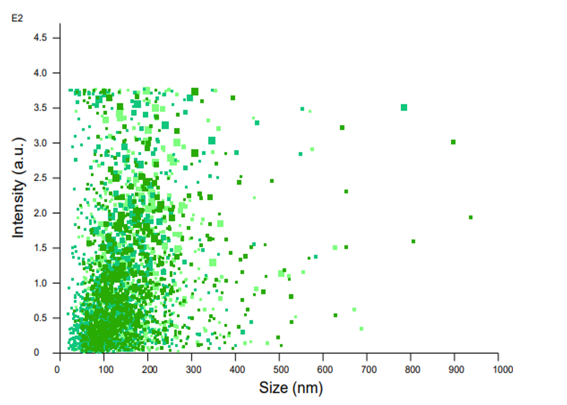

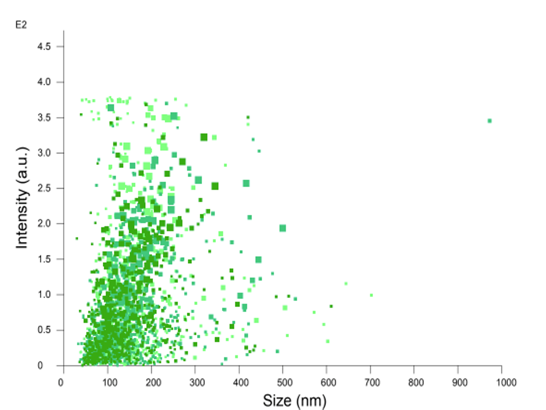


G H


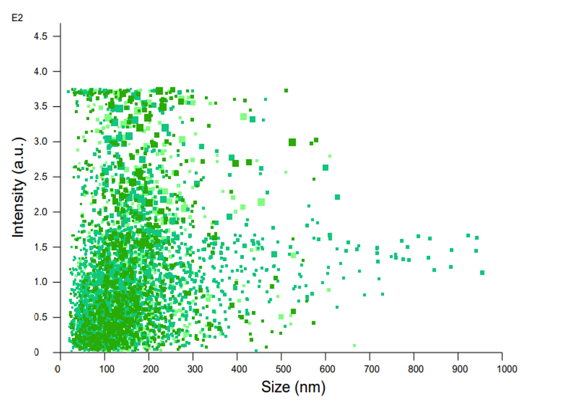

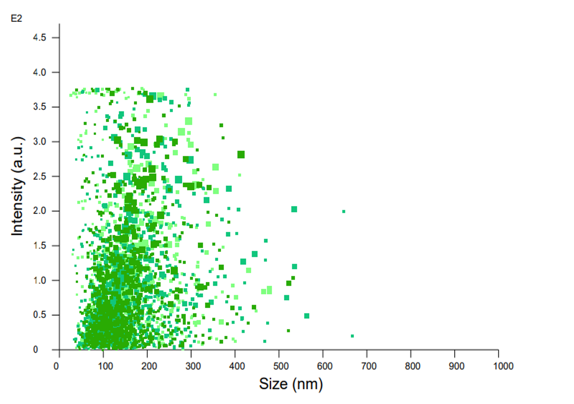


Figure S1: Nanoparticle tracking analysis using NanoSight LM10 system confirmed the presence of EV. Figures A-D represent patient samples, and figures E-H represent health control samples.

Supplementary Videos S1-8 are separately attached to the manuscript submission. Legend for those videos is as below.

Supplementary Videos S1-8: 8 representative videos (10 s duration each) of Brownian motion of nanoparticles were recorded and analyzed by NanoSight LM10. Videos S.A-D represent patient samples and samples videos S E-H represent healthy control samples.
