# Supplementary material for: Author Correction: Circulating microRNA-155-3p levels predicts response to first line immunotherapy in patients with metastatic renal cell carcinoma
Source: Sci Rep. 2025 Apr 28;15:14900. doi: 10.1038/s41598-025-99376-z (PMC12037859; doi:10.1038/s41598-025-99376-z)
Supplement: Supplementary file 1 — Supplementary Information. [file 41598_2025_99376_MOESM1_ESM.docx]

**SUPPLEMENTARY FIGURES**

Supplementary Table S1: specific miRNA which were implicated in renal cell carcinoma.

| **Specific miRNA** | **Activity** | **Reference** |
| --- | --- | --- |
| miR-1233-3p | Regulation of p53 gene expression | 34 |
| miR-221-5p | Regulation of the EGFR pathway, modulation of dendritic cell maturation | 35–37 |
| miR-200 | PD-L1 expression regulation, development of RCC metastases | 38–40 |
| miR-155-3p | Regulation of T-regulatory cell activity, regulation of AID, regulation of PD-L1 expression, regulation of VHL expression, potential targeting of FOXO3 | 33,41–44 |
| miR-424 | Anti-tumour immune response | 45 |
| miR-138-5p | Immune checkpoint regulation of CTLA-4 and PD-1 | 46,47 |
| miR-497-5p | Associated with upregulation of PD-L1 in mRCC | 48 |
| miR-520c-3p | NK cell activity, IL8 regulation | 49,50 |
| miR-3065-5p | Angiogenic modulation in RCC | 51 |

Supplementary Figure S1:


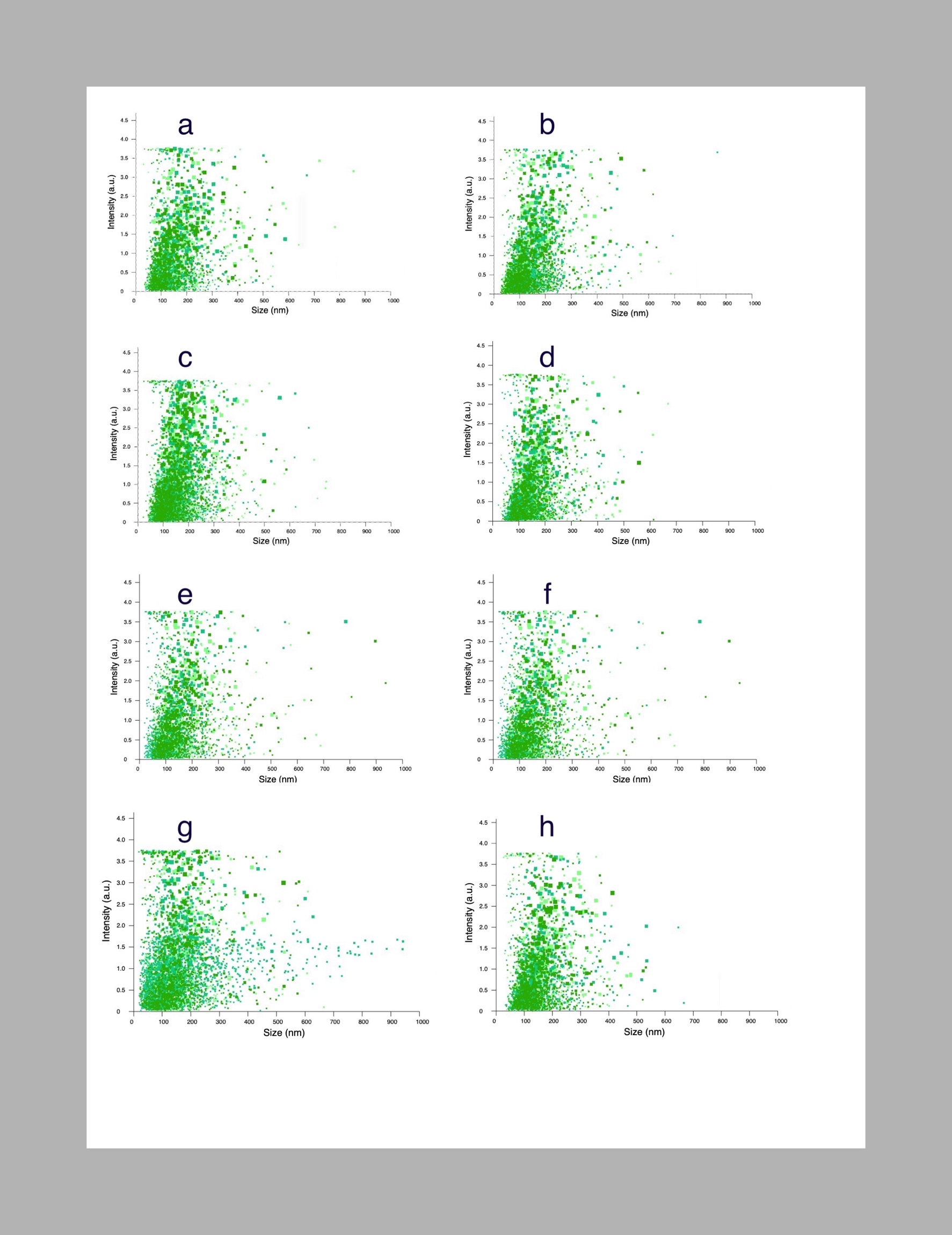


Figure S1: Nanoparticle tracking analysis using NanoSight LM10 system confirmed the presence of EV. Figures A-D represent patient samples, and figures E-H represent health control samples.

Supplementary Videos S1-8 are separately attached to the manuscript submission. Legend for those videos is as below.

Supplementary Videos S1-8: 8 representative videos (10 s duration each) of Brownian motion of nanoparticles were recorded and analyzed by NanoSight LM10. Videos S.A-D represent patient samples and samples videos S E-H represent healthy control samples.
